# Effect of dietary aspirin eugenol ester on the growth performance, antioxidant capacity, intestinal inflammation, and cecal microbiota of broilers under high stocking density

**DOI:** 10.1016/j.psj.2024.103825

**Published:** 2024-05-11

**Authors:** Haojie Zhang, Yi Zhang, Dongying Bai, Jiale Zhong, Xiaodi Hu, Ruilin Zhang, Wenrui Zhen, Koichi Ito, Bingkun Zhang, Yajun Yang, Jianyong Li, Yanbo Ma

**Affiliations:** ⁎ Department of Animal Physiology, College of Animal Science and Technology, Henan University of Science and Technology, Luoyang 471003, China; † Innovative Research Team of Livestock Intelligent Breeding and Equipment, Longmen Laboratory, Luoyang 471023, China; ‡ Henan International Joint Laboratory of Animal Welfare and Health Breeding, College of Animal Science and Technology, Henan University of Science and Technology, Luoyang 471023, China; § Department of Food and Physiological Models, Graduate School of Agricultural and Life Sciences, The University of Tokyo, Ibaraki 319-0206, Japan; # State Key Laboratory of Animal Nutrition, Department of Animal Nutrition and Feed Science, College of Animal Science and Technology, China Agricultural University, Beijing, 100193, China; ǁ Key Lab of New Animal Drug of Gansu Province, Key Lab of Veterinary Pharmaceutical Development of Ministry of Agriculture and Rural Affairs, Lanzhou Institute of Husbandry and Pharmaceutical Science of Chinese Academy of Agricultural Sciences, Lanzhou, 730046, China

**Keywords:** broiler, high stocking density, aspirin eugenol ester, antioxidant capacity, intestinal inflammation, intestinal microbiota

## Abstract

This study was designed to examine the impact of aspirin eugenol ester (**AEE**) on the growth performance, serum antioxidant capacity, jejunal barrier function, and cecal microbiota of broilers raised under stressful high density (**HD**) stocking conditions compared with normal density broilers (**ND**). A total of 432 one-day-old AA+ male broilers were randomly divided into 4 groups: normal density (ND, 14 broilers /m^2^), high density (**HD**, 22 broilers /m^2^), ND + AEE, and HD + AEE. The results of the study revealed a significant decrease in the growth performance of broiler chickens as a result of HD stress (*P* < 0.05). The total antioxidant capacity (**T-AOC**) in serum demonstrated a significant decrease (*P* < 0.05) at both 28 and 35 d. Conversely, the serum level of malondialdehyde (**MDA**) exhibited a significant increase (*P* < 0.05). Dietary supplementation of AEE resulted in a significant elevation (*P* < 0.05) of serum GSH-PX, SOD and T-AOC activity at both 28 and 35 d. Moreover, exposure to HD stress resulted in a considerable reduction in the height of intestinal villi and mRNA expression of tight junction proteins in the jejunum, along with, a significant elevation in the mRNA expression of inflammatory cytokines (*P* < 0.05). However, the administration of AEE reversed the adverse effects of HD-induced stress on villus height and suppressed the mRNA expression of the pro-inflammatory genes, *COX-2* and *mPGES-1*. Additionally, the exposure to HD stress resulted in a substantial reduction in the α-diversity of cecal microbiota and disruption in the equilibrium of intestinal microbial composition, with a notable decrease in the relative abundance of *Bacteroides* and *Faecalibacterium* (*P* < 0.05). In contrast, the addition of AEE to the feed resulted in a notable increase in the relative abundance of *Phascolarctobacterium* and enhanced microbial diversity (*P* < 0.05). The inclusion of AEE in the diet has been demonstrated to enhance intestinal integrity and growth performance of broilers by effectively mitigating disruptions in gut microbiota induced by HD stress.

## INTRODUCTION

Over the course of many years, the global broiler industry has exhibited a pattern of steady expansion, culminating in a staggering global production volume of 102.259 million tons in 2023 (Livestock and Poultry, 2023). The global broiler industry faces challenges in maintaining profitability due to the elevated prices of feed and energy. To enhance economic and production efficiency, breeders resort to intensive farming methods, increasing breeding density and scaling up production (Pesti and Choct, 2023). In the domain of meat and poultry breeding, feeding density can be quantified either by the number of animals or the weight of the field per unit feeding area, denoted as broilers/m^2^ or kg/m^2^. Based on contemporary feeding standards that consider factors such as animal welfare, growth performance, and various other considerations, a density of 16 broiler/m^2^ or 39 kg/m^2^ is deemed acceptable (Palizdar et al*.,* 2017; Son et al*.,* 2022). In pursuit of economic benefits, however, there is a tendency to opt for higher stocking densities (**HD**). Although this strategy can reduce breeding expenses and increase profits, it can also result in changes to the behavior, physiology, and metabolism of broilers, ultimately triggering stress responses (Jones et al*.,* 2005; Najafi et al*.,* 2015; Evans et al*.,* 2023). The stressors linked to HD stress exhibit a significantly intricate nature in comparison to other stress reactions.

The occurrence of HD stress is linked to increased temperatures in the chickens’ microenvironment and the accumulation of noxious gases in their growth environment, which leads to oxidative stress and reduced growth performance. When the equilibrium between oxidation and antioxidant processes is disturbed, it causes an increase in the level of reactive oxygen species (**ROS**) within the chicken's body, resulting in oxidative damage (Surai et al*.,* 2019; Son et al*.,* 2022). There is an increasing body of evidence suggesting a robust correlation between oxidative stress and the adverse outcomes encountered in poultry production (Mao et al*.,* 2024). The serum catalase (**CAT**), total antioxidant capacity (**T-AOC**), superoxide dismutase (**SOD**), and glutathione peroxidase (**GSH-PX**) are the chief components of the antioxidant defense mechanism (Kong et al*.,* 2022). Prior studies have confirmed that HD stress from elevated feeding density can have a negative influence on various serum parameters, resulting in heightened oxidative harm to broilers (Li et al*.,* 2019; Humam et al*.*, 2020).

The gut, as an organ of the body, has the function of digestion and absorption, while also playing a crucial role in guarding the animal against harmful intestinal bacteria and toxins (Kulkarni et al*.,* 2023). The negative impact of HD stress on the well-being of the intestinal microbiota and the mucosal integrity of broilers has been established, and our present study expands upon this by showing that HD stress significantly downregulates the expression of essential tight junction proteins, such as *occludin*, c*laudin-1*, and *ZO-1*, in the intestinal mucosal barrier (Liu et al*.,* 2023c; Li et al*.*, 2023). Intestinal barrier impairment is commonly accompanied by inflammation, and research has demonstrated that HD stress can induce necrotizing enteritis in broilers, augment the secretion of pro-inflammatory cytokines such as *IL-1β, IL-6*, and *TNF-α*, impair the digestion, and hinder feed digestibility and nutrient absorption in broilers (Camilleri et al*.,* 2012; Tsiouris et al*.,* 2015; Li et al., 2019). Cyclooxygenase-2 (***COX-2***) is closely related to the inflammatory response. *COX-2* catalyzes the production of prostaglandin G2 from arachidonic acid which is then converted to prostaglandin H2, and ultimately produces prostaglandin E2 (***PGE2***) from the enzymatic reaction of prostaglandin E synthase (***PGES***) (Ricciotti and FitzGerald, 2011). This cascade of reactions can cause intestinal barrier breakdown, inflammatory bowel disease, and inflammatory response in intestinal epithelial cells (MacDermott, 1994; Lugo et al*.,* 2007).Among the *PGE2* synthases, microsomal prostaglandin E synthase-1 (***mPGES-1***) is upregulated in response to various inflammatory stimuli, and its expression pattern is similar to that of *COX-2* (Riendeau et al*.,* 2005). At the same time, HD stress decreased the villus height in the jejunum and significantly changed the composition of intestinal bacteria. HD stress increased the number of *Enterococcus faecalis* and decreased the number of *Lactobacillus* and *Bifidobacterium* (Li et al*.,* 2017; Wu et al*.,* 2019). These changes in the intestinal microbiota can reduce the efficiency of digestion and nutrient absorption and have a negative impact on poultry production (Sugiharto and Ranjitkar, 2019).

Aspirin eugenol ester (**AEE**) is a novel chemical compound synthesized by esterification of aspirin and eugenol. It is well known that aspirin, which has been widely used for more than 100 y, still plays an important role in clinical practice due to its analgesic, antipyretic and anti-inflammatory properties. Its mechanism of action is related to the irreversible inhibition of the cyclooxygenase enzyme involved in prostaglandin synthesis. It has anti-inflammatory, antiviral, antibacterial, and antioxidant properties and can significantly improve gut health (Taleuzzaman et al*.,* 2021). Nevertheless, clinical studies have indicated that aspirin can lead to substantial harm to the gastrointestinal tract. Eugenol also has some drawbacks such as strong odor and susceptibility to oxidation, which originates from the reaction between the carboxyl group in the aspirin and a hydroxyl group in eugenol (Suyama et al*.,* 2018; Rasul et al*.,* 2021). AEE not only manifests a lower level of toxicity and an extended duration of action, but also possesses a broad safety range as a highly therapeutic compound without the unfavorable properties associated with the prodrug (Li et al*.,* 2012). Investigations of the impact of AEE on proteins associated with inflammation and inflammatory factors, the concentration of cyclooxygenase-1 (***COX-1***) and *COX-2* in rat serum was significantly reduced by AEE, and the expression of *IL-1β, IL-6,* and *TNF-α* in vivo and in vitro was decreased (Ma et al*.*, 2017; Liu et al*.,* 2023b).The evaluation of the effects of AEE on the metabolomics and microbiota of cecum contents in hyperlipidemic rats demonstrated that AEE successfully reinstated normal metabolic and digestive function through modifications in the composition of the intestinal microbiota (Ma et al*.,* 2018).

Most of the recent research studies on the mechanism of AEE action have involved cultured cells or mice, with only a limited number of investigations conducted on livestock and poultry. There is a dearth of knowledge regarding the effect of AEE on intestinal barrier integrity and inflammation in broilers and the lack of understanding of the underlying mechanism emphasizes the need of further exploration within the context of the HD stress model. Consequently, the objective of this investigation was to determine the nature of AEE's effect on growth performance, antioxidant metabolism, jejunal gene expression, and the cecal microbiota in broilers under HD-induced stress.

## MATERIALS AND METHODS

The Experimental Animal Care and Utilization Committee of Henan University of Science and Technology granted approval to the experimental protocol employed in this study (AW20602202-1-2), which was conducted in accordance with the Guidelines for Experimental Animals issued by the Ministry of Science and Technology (Beijing, China).

### Animals and Experimental Design

A total of 432 one-day-old male AA+ broilers, with good health and similar body weight (Henan Quanda Poultry Breeding Company Co., Ltd., Henan, China) were randomly divided into 4 treatment groups: (1) normal density (**ND**) at 14 broilers/m^2^ fed the basal diet; (2) high density (**HD**) at 22 broilers/m^2^, fed the basal diet; (3) normal density +AEE (**ND+AEE**) at 14 broilers/m^2^ fed the basal diet + 0.01% AEE; (4) high density +AEE (**HD+AEE**), 22 broilers/m^2^ fed the basal diet + 0.01% AEE. There were 12 replicates per treatment with 7 broilers per replicate in the ND group and 11 broilers per replicate in the HD group. The experimental period was 42 d. AEE was prepared by Lanzhou Institute of Animal Science and Veterinary Medicine, Chinese Academy of Agricultural Sciences with a purity of > 99.5%. The choice of AEE concentration employed in this study was based on our prior unpublished research, which revealed that treatment with 0.01% AEE resulted in optimal antioxidant activity. The experiment was conducted at the animal test farm affiliated with Henan University of Science and Technology. Prior to the commencement of the experiment, thorough cleaning and disinfection of the chicken coop and feeding equipment were carried out in strict adherence to established protocols. The broilers were housed in a mechanically ventilated room, specifically designed with a stacked 3-dimensional cage system. The broilers were provided with unrestricted access to food and water, and their experimental diets were divided into 2 distinct stages: 1 to 21 d of age and 22 to 42 d of age, as outlined in Table 1**.** During the initial stage (d 1–5), the temperature within the feeding room was maintained at 32 to 34°C, with weekly reductions of 2°C until it reached the final range of 22 to 24°C. The relative humidity was consistently maintained at 40 to 60%. For the first 3 d, the broilers were exposed to continuous light, followed by a subsequent schedule of 23 h of light and 1 h of darkness. The housing area was frequently sanitized and the animals were provided with optimal nourishment and immunization, with vigilant monitoring and accurate documentation of the growth process.

**Table 1:** Basic diet formula and nutrient levels.

Ingredient	Content (%)	
	1–21d	22–42d
Corn	52.79	57.78
Soybean meal	36.89	30
Zea gluten meal	0	2.43
Soybean oil	4	4
Sodium chloride	0.3	0.3
Choline chloride	0.3	0.26
Vitamin premix	0.03	0.03
Trace element premix	0.2	0.2
Stone powder	1.222	1.171
Dicalcium phosphate	1.912	1.623
DL-Methionine	0.265	0.106
L-Lysine	0.038	0.045
Wheat bran	2	2
Total	100	100
Metabolizable energy (MJ/kg)	12.4	13.0
Crude protein	211.8	198.4
Lysine	11.4	10.5
Methionine	4.9	4.8
Calcium	10.2	8.5
Available P	4.5	4.2
Total P	6.9	6.3
Threonine	7.7	2.2
Analyzed content		
Calcium	10.2	8.5
Calcium	6.8	6.2
Calcium: Total P	1.50	1.37

The premix of trace elements provided copper, 8 mg (CuSO4·5H_2_O) per kg of formula feed; iron, 80 mg (FeSO_4_); manganese, 100 mg (MnSO_4_·H_2_O); selenium, 0.15 mg (Na_2_SeO_3_); Iodine 0.35 mg (KI). A vitamin premix was provided at the indicated amount per kg of formula feed: VA 9500 IU; VD3 62.5 µg; VE 30 IU; VK3 2.65 mg; VB1 2 mg; VB6 6 mg; VB12 0.025 mg; biotin 0.0325 mg; folic acid 1.25 mg; pantothenic acid 12 mg; and niacin 50 mg.

### Growth Performance Measurement and Sample Collection

Throughout the duration of the experiment, at 21, 28, 35, and 42 d of age, the body weight and feed intake were documented after an 8-h period of feed deprivation. Subsequently, the average daily gain (**ADG**), average daily feed intake (**ADFI**), and feed conversion rate (**FCR**) were computed for each group. The progress of broiler growth was measured and recorded daily, the chicken was found to be dead, and the time of chicken death, the remaining amount of feed and the weight of the chicken were recorded at the first time, and the influence of chicken death on the experimental data was eliminated in the subsequent process of experimental data processing. Six broilers were randomly selected from 6 replicates at 21, 28, 35 and 42 d of age in each group. Blood samples were collected from the wing vein, allowed to clot, and the serum was separated by centrifuging at 4°C and 3,000 rpm for 10 min and subsequently stored in a refrigerator at -80°C for further testing. Animals in each treatment group are randomly selected and euthanized by inhalation of carbon dioxide. The middle section of the jejunum was removed, and one part was rinsed with cold normal saline and fixed in 4% paraformaldehyde tissue for sectioning and microscopic examination of intestinal morphology after staining with hematoxylin and eosin (**H&E**). The other part was frozen in liquid nitrogen and stored at -80°C. The cecum was removed the cecum digesta were collected, placed in an RNA-free enzyme tube, immediately followed by storing it in a liquid nitrogen tank at -80°C for subsequent determinations.

### Determination of Serum Antioxidant Capacity

The serum malondialdehyde (**MDA**) concentration was determined using the thiobarbituric acid (**TBA**) method following kit instructions. The assessment of serum glutathione peroxidase (**GSH-PX**) and superoxide dismutase (**SOD**) activity was carried out through a colorimetric approach and the WST-1 method, respectively. Lastly, the measurement of serum total antioxidant capacity (**T-AOC**) was conducted utilizing the ABTS method. These assays were performed utilizing kits (A-003-1-2, A-005-1-2, A-001-3-2, and A-015-2-1) from the Nanjing Jiancheng Bioengineering Institute Co., Ltd. (Nanjing, China).

### Intestinal Morphology

The jejunal tissue samples were fixed in 4% paraformaldehyde, dehydrated in a graded series of ethanol solutions (50, 70, 80, 90, 95, and 100%), embedded in paraffin, and sectioned. Subsequently, xylene was used to remove the wax, and the samples were rehydrated and stained following a published method (Rieger et al*.,* 2021). The stained sections were then visualized using a scanner (Panoramic MIDI, Hungary) for evaluation of intestinal morphology and anatomical measurements. Ten well-oriented, intact villi were selected for measurement using CaseViewer software (version 2.0). Villus height, defined as the distance from the tip of the villus to the opening of the villus recess, and crypt depth, the distance from the base of the intestinal gland to the opening of the crypt, were measured. The ratio of villus height to crypt depth was calculated based on these measurements (De Grande et al*.,* 2020).

### Extraction of Total RNA From the Intestine and Quantitative Real-Time PCR

The jejunal tissue samples were thawed on ice, and 50 to 100 mg of tissue was transferred into a sterile 1.5 mL Eppendorf centrifuge tube. TRIzol (Invitrogen Inc., Carlsbad, CA) reagent was used to extract total RNA from each sample. The RNA was analyzed using a Nanodrop2000 (Thermo Scientific, Ottawa, Canada), and the A260/A280 ratio was >1.9, indicating good purity. The concentration was determined from the absorbance readings and the RNA was aliquotted and stored at -80°C until used for cDNA synthesis. Following the guidelines of the reverse transcription kit (Vazyme, Nanjing, China), the total RNA within the extracted sample was reverse-transcribed into cDNA. The primers, which were designed using Prime 3.0 and synthesized by Shanghai Shenggong Bioengineering Co., LTD., included GAPDH as the reference gene (Table 2). Real-time fluorescence quantitative PCR was performed on the CFX Connect Real-Time PCR detection system employing a SYBR Green PCR kit (Vazyme Biotechnology Co., Ltd., Nanjing, China). The reaction conditions were: initial denaturation at 95°C for 5 min, followed by 40 cycles of denaturation at 95°C for 15 s and annealing at 60°C for 30 s. The results of gene expression were analyzed and compared using the 2^−∆∆CT^ method.

**Table 2:** Primer sequences.

Gene^1^	Primer sequence (5′-3′)^2^	Length (nt)	GenBank number
OCLN	F: ACGGCAGCACCTACCTCAA R: GGGCGAAGAAGCAGATGAG	123	XM_025144247.2
CLDN1	F: CATACTCCTGGGTCTGGTTGGT R: GACAGCCA TCCGCA TCTTCT	100	NM_001013611.2
CLDN2	F: CCTACATTGGTTCAAGCATCGTG R: GATGTCGGGAGGCAGGTTGA	131	NM_001277622.1
ZO-1	F: CTTCAGGTGTTTCTCTTCCTCCTC R: CTGTGGTTTCA TGGCTGGATC	144	XM_021098886
COX-2	F: CCGAATCGCAGCTGAATTCA R: GAAAGGCCATGTTCCAGCAT	116	NM_001277664.2
mPGES-1	F: AGGCTCAGGAAGAAGGCATT R: CACAGCTCCAAGGAAGAGGA	153	NM_001194983.1
TNF-α	F: GAGCGTTGACTTGGCTGTC R: AAGCAACAACCAGCTA TGCAC	176	NM_214022.1
IL-1β	F: ACTGGGCA TCAAGGGCTA R: GGTAGAAGA TGAAGCGGGTC	154	NM_214005.1
IL-10	F: AGAAATCCCTCCTCGCCAAT R: AAATAGCGAACGGCCCTCA	121	NM_001004414.2
IL-6	F: GCTGCGCTTCTACACAGA R: TCCCGTTCTCA TCCA TCTTCTC	203	NM_204628.1
GAPDH	F: TGCTGCCCAGAACATCATCC R: ACGGCAGGTCAGGTCAACAA	142	NM_204305

1 TNF-α = tumor necrosis factor-α; IL-1β = interleukin-1β; IL-6 = interleukin-6; IL-10 = interleukin-10; COX-2 = cyclooxygenase-2; mPGES-1 = microsomal prostaglandin E synthase-1; GAPDH = glyceraldehyde-3-phosphate dehydrogenase.

2 F: forward primer; R: reverse primer.

### Cecum Microbial 16S rRNA Sequencing

To assess the intestinal microbial community, total genomic DNA was extracted from cecal contents (100 mg) using the E.Z.N.A. DNA kit (Omega Bio-Tek, Norcross, GA). The quality of the extracted genomic DNA was visualized through 1% agarose gel electrophoresis. The DNA concentration and purity were determined using a NanoDrop2000 nucleic acid analyzer (Thermo Scientific). Three replicates per sample were run to amplify the V3 to V4 variable region of the 16S rRNA gene via PCR. The extracted DNA was utilized as the template, employing 338F (5 '-ACTCCTACGGGAGGCAGCA G-3′) as the upstream primer and 806R (5 '-GGACTACHVGGGTWTCTAAT-3′) as the downstream primer. The PCR products obtained from the sample replicates were combined, separated on a 2% agarose gel, excised and purified with the AxyPrep DNA gel extraction kit (Axygen Biosciences, Union City, CA). The recovered products were then assessed by agarose gel electrophoresis and quantified using the Quantus Fluorometer (Promega) (Liu et al*.,* 2016).

The sequencing process was executed employing a NovaSeq PE250 platform from Illumina (Shanghai Meiji Biomedical Technology Co., Ltd.). Analysis of MiSeq sequencing data was based on our previous report (Liu et al*.,* 2023c). Briefly, the Illumina platform was utilized for processing the initial sequencing data. The DADA2 method was employed to cluster high-quality sequences into operational taxonomic units (**OTUs**) using a 97% similarity threshold. This process involved several steps such as de-priming, quality filtering, de-noising, splicing, and de-chimera detection. The resulting OTUs were annotated and classified based on the Green Genes database. QIIME2 (2019.4), along with Python scripts, was employed to extract and overlap OTUs, perform clustering, calculate alpha-diversity, and assess beta-diversity. To determine the differential abundance of taxa, the linear discriminant analysis effect size (**LEfSe**) was employed. A UPGMA clustering heatmap was generated to analyze the microbial communities, focusing on the top 20 genera based on their abundance. The sample FASTQ information was submitted to the NCBI (Accession No: PRJNA1060885).

### Statistical Analysis

Each cage-replicate was designated as an experimental unit for the purpose of evaluating growth-performance. The individual animals within each replicate served as the experimental units for other analyses. Statistical analysis was conducted using one-way ANOVA with SPSS20.0 (SPSS Inc., Chicago, IL). Significance between groups was assessed using Tukey's multiple-range test, and significance was defined as *P* < 0.05. The figures and Pearson's correlations were generated using GraphPad Prism 9 (GraphPad Software Inc., San Diego, CA).

## RESULTS

### Effects of AEE on Growth Performance of HD Broilers

The effects of AEE on the growth performance of HD broilers are shown in Table S1. At 0 to 21 d, the ADFI in the HD group was significantly decreased (*P* < 0.05) compared with the ND group; BW, ADG and FCR were not different among groups. At 22 to 42 d, compared with the ND group, BW and ADG in the HD group were significantly decreased *(P* < 0.05), FCR was significantly increased (*P* < 0.05), and ADFI showed no difference. At the same time, compared with the HD group, BW and ADG in the HD+AEE group were significantly increased, and FCR was significantly decreased (*P* < 0.05). There were no significant differences in BW, ADFI, ADG, and FCR between the ND+AEE group and the ND group. Therefore, we performed a detailed analysis of 22 to 42 d of age, and the results are shown in Table 3. At 22 to 28 d, there were no differences in BW, ADFI, ADG, and FCR in the HD and ND+AEE groups compared with the ND group, and there were no differences in ADFI, BW, ADG and FCR in the HD+AEE group compared with the HD group. At 29 to 35 d, compared with the ND group, BW and ADG were significantly decreased and FCR was significantly increased in the HD group (*P* < 0.05), and BW and ADG and FCR were significantly increased in the HD+AEE group compared with the HD group (*P* < 0.05). At 36 to 42 d, compared with the ND group, the BW, ADFI and ADG in the HD group were significantly decreased, and the FCR was significantly increased (*P* < 0.05), while the BW in the HD+AEE group was significantly increased compared with the HD group (*P* < 0.05).

**Table 3:** Effect of AEE on growth performance of HD broilers.

Items	Group				SEM	*P*-value
	ND	HD	ND+AEE	HD+AEE		
1–21d						
BW,g	654.70	634.22	651.69	639.67	4.69	0.38
ADFI,g	41.02^ab^	39.57^b^	41.86^a^	40.42^ab^	0.35	0.02
ADG,g	31.18	30.20	31.03	30.46	0.22	0.38
FCR	1.32	1.31	1.35	1.33	0.01	0.48
22–28d						
BW,g	1186.45^ab^	1130.07^b^	1240.93^a^	1197.37^ab^	11.85	0.09
ADFI,g	116.13^bc^	115.11^ab^	120.13^b^	121.97^a^	0.93	0.02
ADG,g	70.00^ab^	66.54^b^	75.28^a^	73.86^ab^	1.32	0.07
FCR	1.66	1.74	1.60	1.65	0.03	0.24
29–35d						
BW,g	1880.70^a^	1604.14^b^	1870.26^a^	1769.31^a^	15.06	<0.01
ADFI,g	160.52^a^	145.00^ab^	152.23^ab^	139.37^b^	2.90	0.04
ADG,g	99.18^a^	71.06^c^	89.91^b^	81.71^b^	2.54	<0.01
FCR	1.64^b^	1.99^a^	1.70^b^	1.71^b^	0.05	<0.01
36–42d						
BW,g	2527.83^a^	2325.28^c^	2531.49^a^	2406.08^b^	22.07	<0.01
ADFI,g	197.47^a^	188.07^b^	193.92^ab^	188.61^ab^	1.57	0.09
ADG,g	111.30^a^	94.31^c^	106.17^ab^	98.99^bc^	2.07	0.01
FCR	1.79^b^	2.01^a^	1.83^b^	1.88^ab^	0.03	0.02

ND, normal stocking density fed basal diet; HD, high stocking density fed basal diet; ND+AEE normal stocking density fed basal diet supplemented with 0.01% AEE; HD+AEE high stocking density group fed basal diet supplemented with 0.01% AEE. BW, body weight; ADG, average daily gain; ADFI, average daily feed intake; FCR, feed conversion ratio (feed: gain, g: g).

a,b,c Means within a row with no common superscript differ significantly (n = 12, *P* < 0.05)

### Effects of AEE on Serum Antioxidant Capacity of HD Broilers

**Figure 1:**
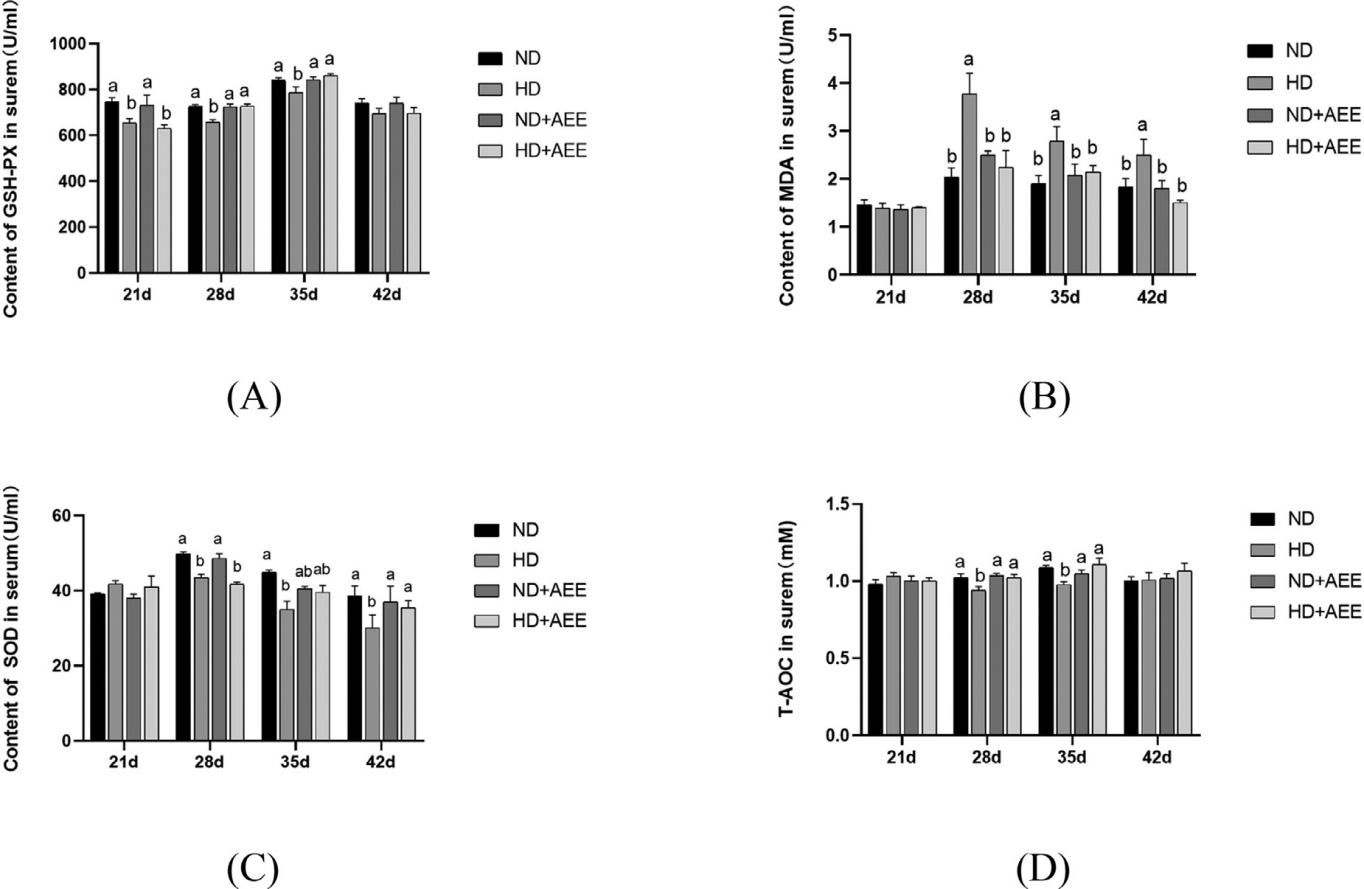
Effects of AEE on serum antioxidant parameters of broilers under HD stress. (A) glutathione peroxidase (**GSH-PX**). (B) malondialdehyde (**MDA**); (C) superoxide dismutase (**SOD**); (D) total antioxidant capacity (**T-AOC**). ND, normal stocking density fed basal diet; HD, high stocking density fed basal diet; ND+AEE normal stocking density fed basal diet supplemented with 0.01% AEE; HD+AEE high stocking density group fed basal diet supplemented with 0.01% AEE. Bars with different letters (a, b) differ significantly across all groups (n = 6, *P* < 0.05).

The effects of AEE on serum antioxidant indexes of HD broilers are shown in Figure 1. Compared with the ND group, the serum GSH-PX content in the HD group at 21, 28, and 35 d of age was significantly decreased (*P* < 0.05). Compared with the HD group, dietary supplementation with AEE in the HD+AEE group significantly increased (*P* < 0.05) serum GSH-PX content at 28 and 35 d of age (Figure 1A). Compared with the ND group, the MDA content in the HD group was significantly increased (*P* < 0.05) at 28, 35 and 42 d of age, and the SOD content in the HD group was significantly decreased (*P* < 0.05) at the same days of age. Dietary supplementation with AEE significantly decreased (*P* < 0.05) the serum MDA content in the HD+AEE group at 28, 35, and 42 d of age, while significantly increasing serum SOD (Figures 1B and 1C), compared with the untreated HD group. Compared with the ND group at 28 and 35 d of age, the serum MDA content in the HD+AEE groups was significantly decreased (*P* < 0.05). The serum T-AOC of broilers in the HD group was significantly decreased (*P* < 0.05) relative to ND, and the serum T-AOC of broilers in the HD+AEE group was significantly increased relative to the HD group (*P* < 0.05) (Figure 1D).

### Effect of AEE on Jejunum Morphology of HD Broilers

**Figure 2:**
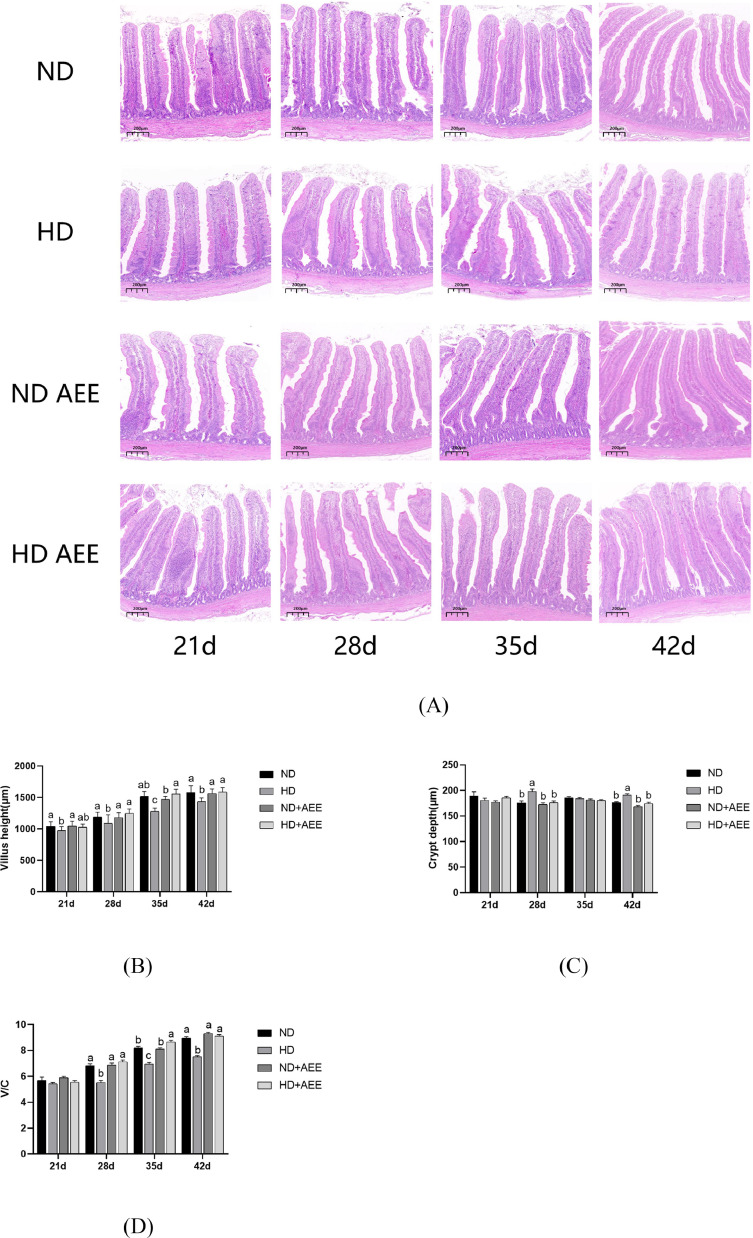
Effects of AEE on jejunal morphology of broilers under HD stress. (A) Morphological structure of jejunum at 21, 28, 35 and 42 d. Scale bar = 100 mm. (B) Average villus height in jejunal intestine. (C) Crypt depth of jejunal intestine. (D) V/C, ratio of villus height to crypt depth in jejunal intestine. ND, normal stocking density fed basal diet; HD, high stocking density fed basal diet; ND+AEE normal stocking density fed basal diet supplemented with 0.01% AEE; HD+AEE high stocking density group fed basal diet supplemented with 0.01% AEE. Bars with different letters (a,b,c) differ significantly across all groups (n = 6, *P* < 0.05).

As shown in Figure 2A, there is a notable difference in the villus morphology of broilers between the HD and ND groups, with the former exhibiting abnormal characteristics. At d 21, 28, 35 and 42, the jejunal villus height of the HD group was significantly decreased (*P* < 0.05) compared with the ND group, but no significant difference was found relative to the ND+AEE group. On d 28, 35, and 42, the jejunal villus height in the HD+AEE group was significantly higher than that in the HD group (*P* < 0.05) (Figure 2B). On d 28 and 42, the jejunum crypt depth in the HD group was significantly increased (*P* < 0.05) compared with ND, but no significant change was found relative to the ND+AEE group. On d 28 and 42, the jejunal crypt depth in the HD+AEE group was significantly decreased compared with the HD group (*P* < 0.05) (Figure 2C). On d 28, 35 and 42, compared with ND group, the V/C ratio in the HD group was significantly decreased (*P* < 0.05), while the V/C ratio in the ND+AEE group was unchanged. On d 28, 35, and 42, the V/C ratio in the HD+AEE group was significantly higher (*P* < 0.05) than that in the HD group (Figure 2D).

### Effect of AEE on Tight Junction Gene Expression in the Jejunum of HD Broilers

**Figure 3:**
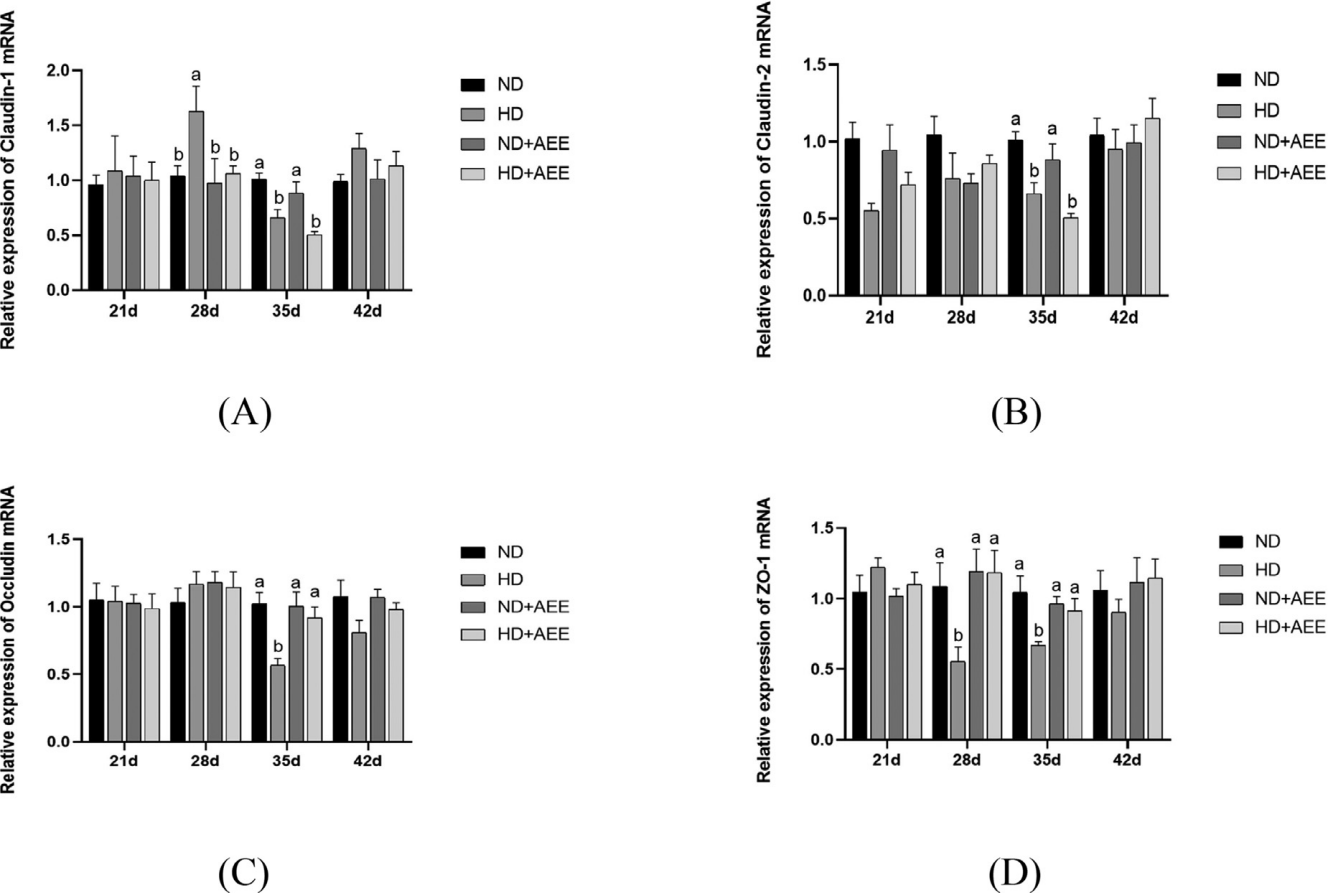
Effect of AEE on the relative expression level of tight junction protein mRNA in the jejunum of HD-stressed broilers. (A) Relative mRNA expression of the jejunal *claudin-1* gene. (B) Relative mRNA expression of the jejunal *claudin-2* gene. (C) Relative mRNA expression of the jejunal *occludin* gene. (D) Relative mRNA expression of the jejunal *ZO-1* gene. ND, normal stocking density fed basal diet; HD, high stocking density fed basal diet; ND+AEE normal stocking density fed basal diet supplemented with 0.01% AEE; HD+AEE high stocking density group fed basal diet supplemented with 0.01% AEE. Bars with different letters (a, b) differ significantly across all groups (n = 6, *P* < 0.05).

The impact of AEE on the maintenance of the intestinal barrier in broiler chickens subjected to HD conditions is depicted in Figure 3. On d 28, the mRNA expression of ZO-1 in the HD group was significantly reduced (*P* < 0.05) compared to the ND group, while the mRNA expression of claudin-1 was significantly elevated (*P* < 0.05). However, the mRNA expression of ZO-1 in broilers from the HD+AEE group was significantly increased (*P* < 0.05) compared to the HD group (Fig. 3A and D). On d 35, the mRNA expression of claudin-1, claudin-2, occludin, and ZO-1 in both the HD and the HD+AEE groups showed significant decreases (*P* < 0.05) compared to the ND group. Moreover, the mRNA expression of occludin and ZO-1 in broilers from the HD+AEE group was significantly increased (*P* < 0.05) compared to the HD group (Figures 3C and 3D).

### Effect of AEE on Inflammatory Gene Expression in the Jejunum of HD Broilers

**Figure 4:**
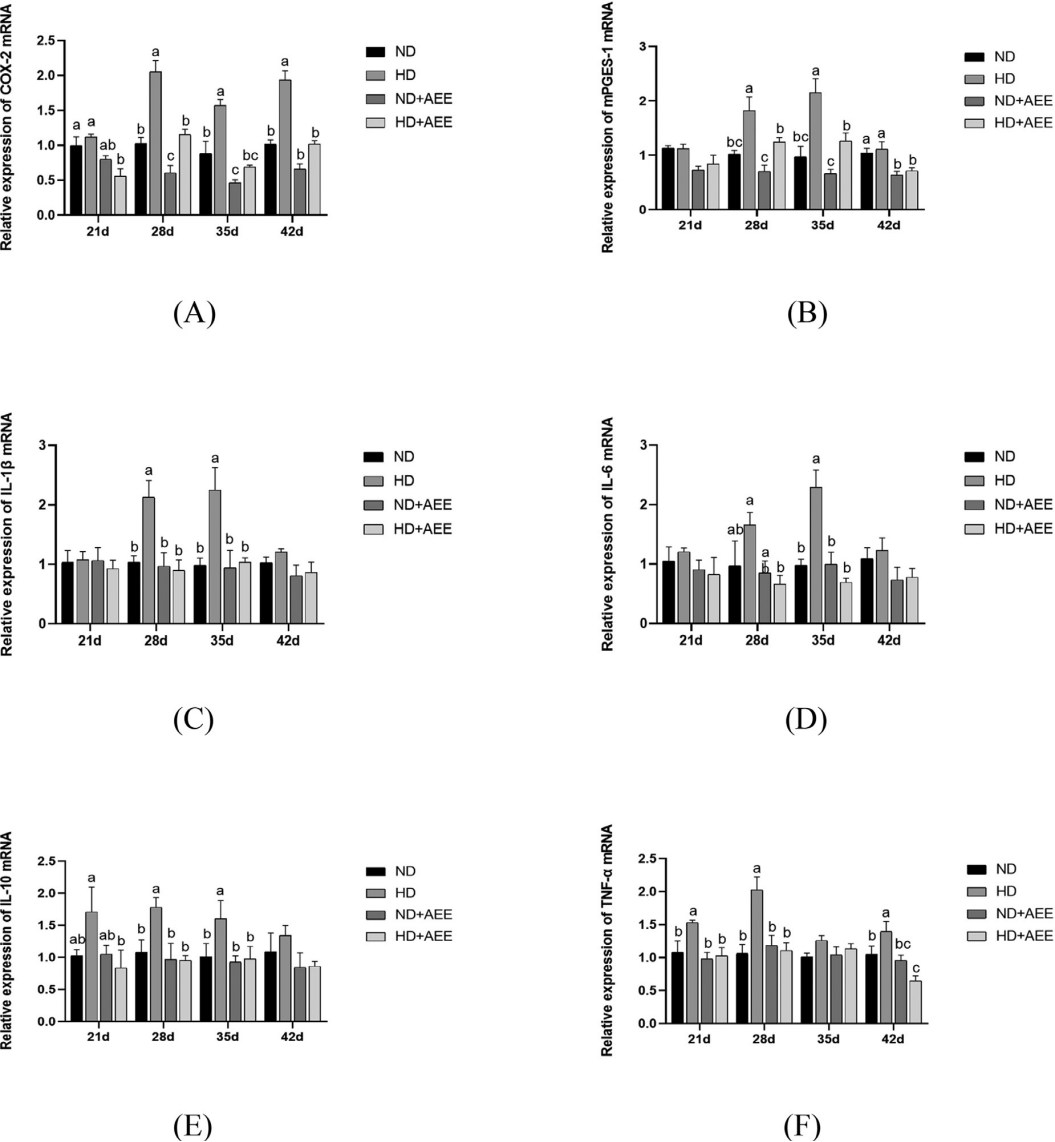
Effect of AEE on the relative expression level of inflammatory factor mRNA in the jejunum of HD-stressed broiler chickens. (A) Relative mRNA expression of the jejunal *COX-2* gene. (B) Relative mRNA expression of the jejunal *mPGES-1* gene. (C) Relative mRNA expression of the jejunal *IL-1β* gene. (D) Relative mRNA expression of the jejunal *IL-6* gene. (E) Relative mRNA expression of the jejunal *IL-10* gene. (F) Relative mRNA expression of the jejunal TNF-α gene. ND, normal stocking density fed basal diet; HD, high stocking density fed basal diet; ND+AEE normal stocking density fed basal diet supplemented with 0.01% AEE; HD+AEE high stocking density group fed basal diet supplemented with 0.01% AEE. Bars with different letters (a, b) differ significantly across all groups (n = 6, *P* < 0.05).

The mRNA expression of inflammatory cytokines in the jejunum of all groups is shown in figure 4. On d 28, 35, and 42, the expression of COX-2 mRNA in the HD group showed a significant increase (*P* < 0.05) compared with the ND group. The expression of COX-2 mRNA in the ND+AEE group was significantly decreased (*P* < 0.05) on d 28 and 35. The addition of AEE to the diet significantly reduced the expression of COX-2 mRNA of broilers in the HD+AEE group (*P* < 0.05) (Figure 4A). On d 28 and 35, the expression of mPGES-1 mRNA in the HD group was significantly increased (*P* < 0.05) compared to the ND group, while the HD+AEE group showed a significant reduction (*P* < 0.05) in mPGES-1 mRNA expression compared to the HD group (Figure 4B). The levels of IL-1β, IL-6, and IL-10 mRNA in the HD group on d 28 and 35 were notably elevated (*P* < 0.05) compared to the ND group. However, the HD+AEE group showed a significant decrease (*P* < 0.05) in expressions of IL-1β, IL-6, and IL-10 mRNA compared to the HD group (Figures 4C–4E). On d 21, 28, and 42, the TNF-α mRNA level in the HD group was significantly increased (*P* < 0.05) compared to the ND group. Conversely, the HD+AEE group demonstrated a significant decrease (*P* < 0.05) in TNF-α mRNA compared to the HD group (Figure 4F).

### Effects of AEE on Cecal Microorganisms of HD Broilers

**Figure 5:**
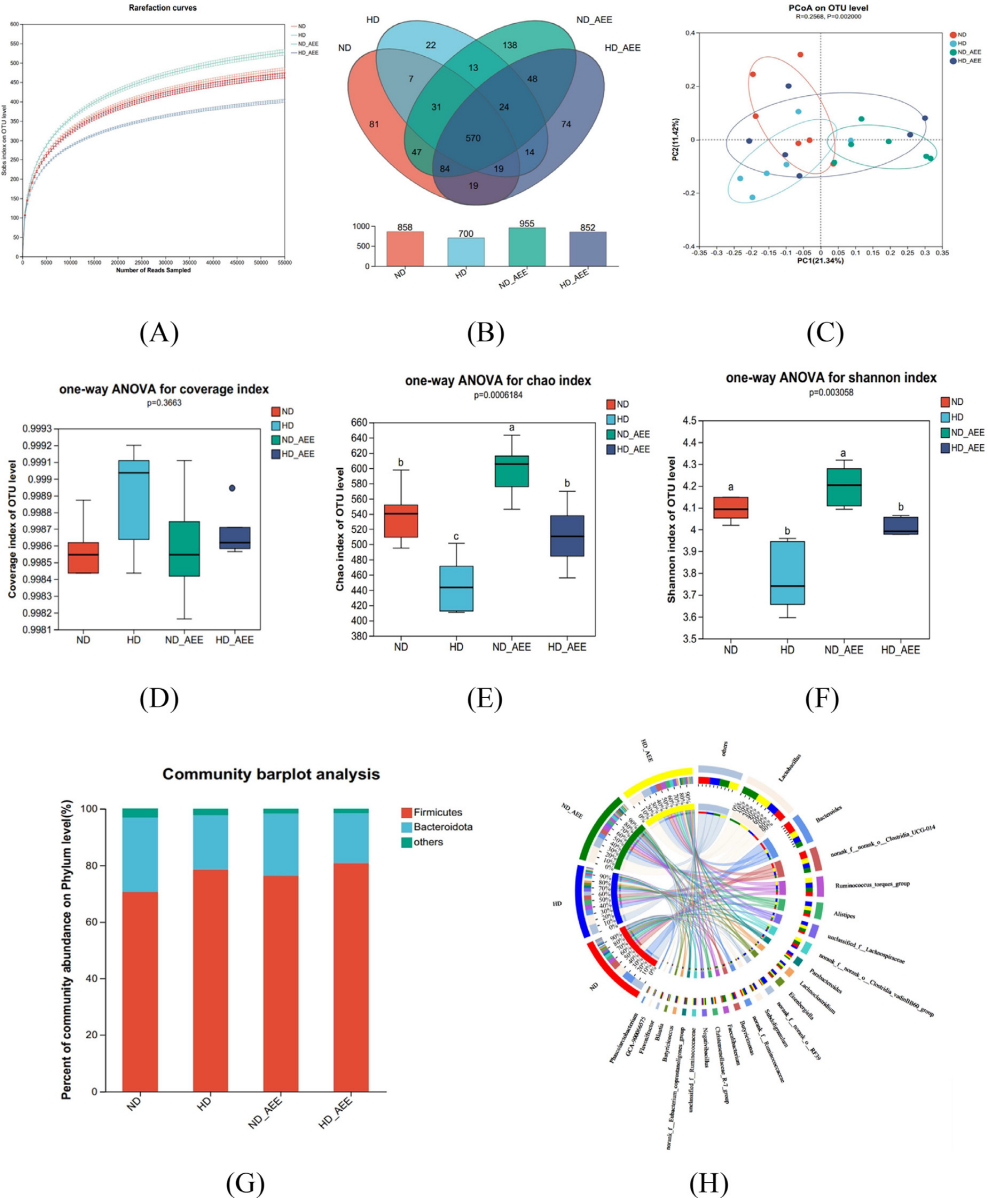

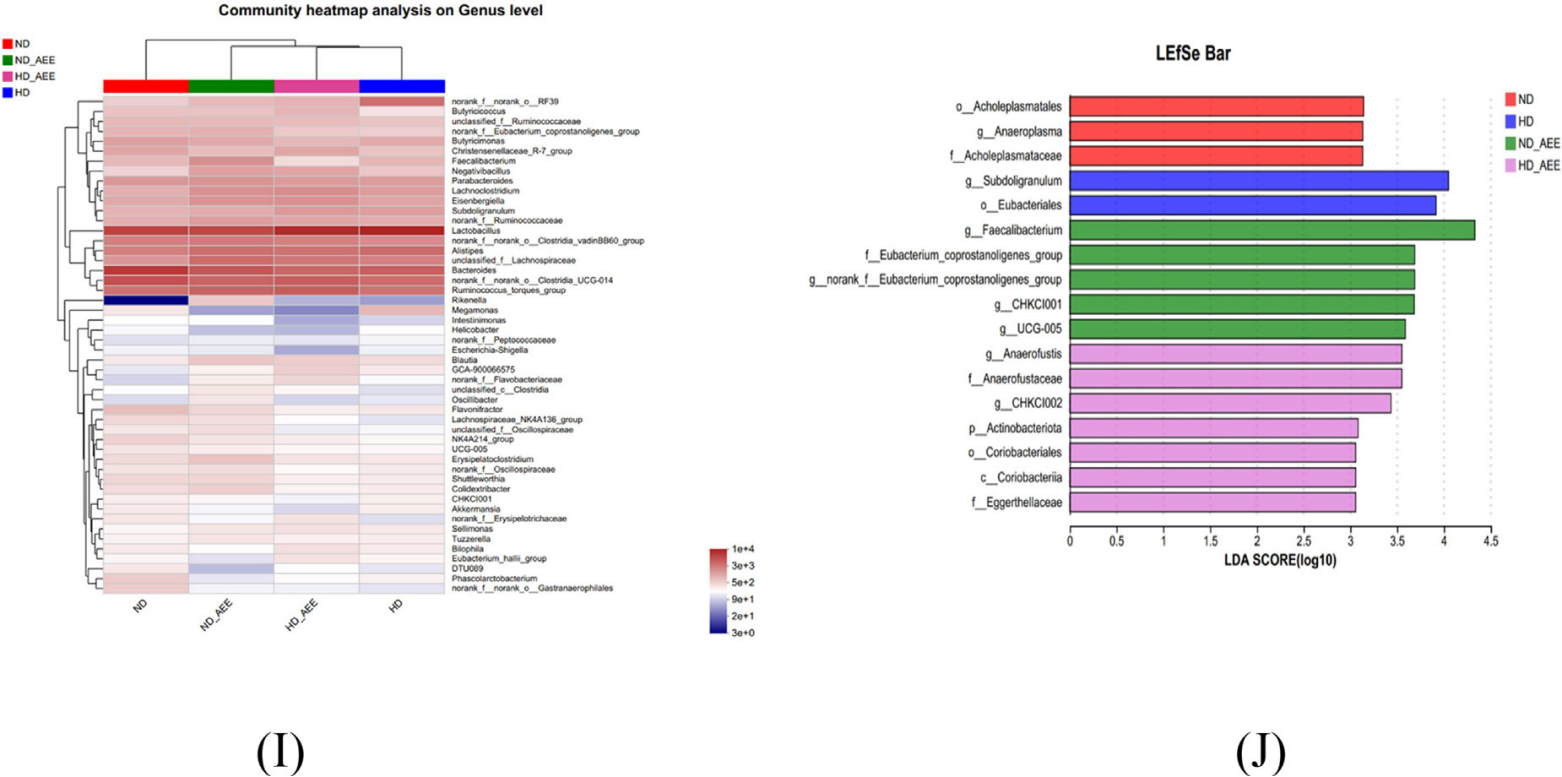
Effect of AEE supplementation on composition and diversity of cecal microbiota of chickens. (A) Rarefaction Curve. (B) Venn diagram based on the OTUs level. (C) Two-dimensional OTU abundance-based principal coordinate analysis (**PCoA**) of cecal microbiota. (D) Coverage index on the OTU level (E–F) AEE was found to increase cecal microbial alpha diversity as measured by the Chao and Shannon indices. (G) Microbial composition at the phylum level. (H) Microbial composition at the genus level. (I) Clustering heatmap of the top 20 genera in each sample. Red represents positive correlation and blue indicates negative correlation. (J) The bar chart obtained through linear discriminant analysis and effect size (LEfSe) analysis shows the differences in microbial abundance in the cecum of broiler chickens. ND, normal stocking density fed basal diet; HD, high stocking density fed basal diet; ND+AEE normal stocking density fed basal diet supplemented with 0.01% AEE; HD+AEE high stocking density group fed basal diet supplemented with 0.01% AEE. Bars with different letters (a,b) differ significantly across all groups (n = 6, *P* < 0.05).

In the investigation of the cecum microbiome, a total of 54,995 high-quality sequences were obtained subsequent to the filtration process. When the sequencing depth surpasses 55,000, the dilution curve approaches saturation, and the coverage index becomes 99.80%, each of which implies that the sequencing data is of substantial magnitude (Figures 5A and 5D). The Venn diagram illustrates the distribution of OTUs within the cecal microbiota of broilers. Based on the diagram, the total count of OTUs within the cecal microbiota in the ND group, HD group, ND+AEE group, and HD+AEE group were recorded as 858, 700, 955, and 582, respectively. Additionally, the number of unique OTUs observed in each of the groups was 81, 22, 138, and 74, respectively (Figure 5B). A β-diversity analysis was conducted to evaluate the degree of similarity in microbiome profiles among the 4 groups.

ANOSIM analysis showed that the microbial composition of the HD group was different from that of the ND group, while HD+AEE restored the gut microbiota of HD broilers to more closely resemble that of the ND group (R = 0.2568, *P* = 0.002) (Figure 5C). Compared with the ND group, the Chao and Shannon indices, which were used to evaluate α diversity, were significantly decreased in the HD group (*P* < 0.05) (Figures 5E and 5F). The statistical analysis conducted on the species composition of cecal microorganisms in the 4 groups revealed that Firmicutes and Bacteroidetes were the prevailing bacterial phyla among all 4 groups (Figure 5G). However, there were no significant differences in the dominant phyla between the 4 groups (Figure S1A). *Lactobacillus, Bacteroides, Clostridia_UCG-014, Ruminococcus torque, Alistipes, Lachnospiraceae, Clostridia_vadinBB6, Parabacteroides, Lachnoclostridium* and *Eisenbergiella* were the dominant taxa in the groups (Figure 5H). In addition, compared with the ND group, the relative abundance of *Bacteroides, Faecalibacterium* and *Phascolarctobacterium* in the HD group was significantly decreased (*P* < 0.05). The relative abundances of *Eisenbergiella, Subdoligranulum* and *GCA-900066575* were significantly increased (*P* < 0.05). Dietary supplementation of AEE increased the relative abundance of *Phascolarctobacterium* and decreased the relative abundance of *Subdoligranulum* in the cecum of the HD+AEE group (Figure S1B), but there was no difference in the other dominant bacterial genera among the 4 groups. Utilizing LEfSe multilevel species difference analysis, bacterial biomarkers were identified to effectively discriminate the differences in cecal microbiota composition among the 4 groups. As shown in Figure 5J, the rare micrococcus *Subdoligranulum* and *Eubacteriales* are enriched in the HD group, and *Faecalibacterium* are enriched in the ND+AEE group. *Anaerofustis, Anaerofustaceae, CHKCI002, Actinobacteriota* and *Coriobacteriales* were enriched in the HD+AEE group. To portray the alterations in species composition and their respective prevalence within each group, a cluster analysis was conducted on the top 50 genera exhibiting the highest relative abundance, followed by the creation of heat maps. The outcomes indicate that the prominent genera observed in the HD group and the HD+AEE group displayed greater similarity in their composition, which notably differed from those observed in the ND group (Figure 5I).

### Correlation Analysis Between Intestinal Microbes and Phenotype

**Figure 6:**
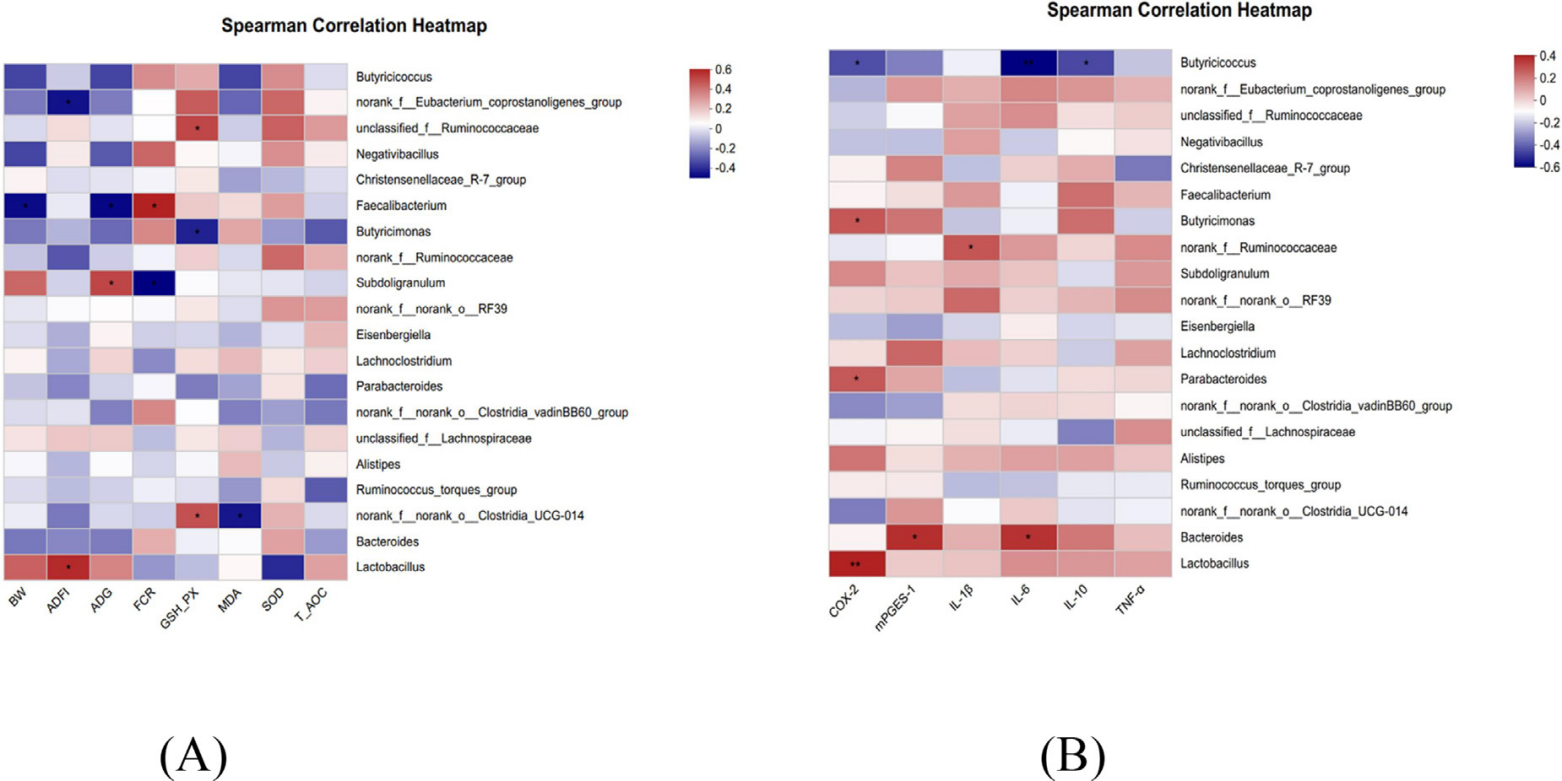
Spearman's correlation analysis between the abundances of intestinal microbiota and phenotypes. (A) Correlation analysis of gut microbiota with growth performance and serum antioxidant capacity. (B) Correlation analysis between intestinal microbiota and intestinal inflammatory indicators. Blue represents a positive correlation and red represents a negative correlation. Significant correlations are noted by 0.01 < *P* ≤ 0.05*, 0.001 < *P* ≤ 0.01**.

The correlation analysis of growth performance, intestinal inflammation and relative abundance of intestinal bacteria at the genus level is shown in Figure 6. The relative abundance of *Lactobacillus* and *Subdoligranulum* was significantly positively correlated with ADG and ADFI. The relative abundance of *Bacteroides, Faecalibacterium* and *Eubacterium coprostanoligenes* was negatively correlated with BW, ADFI, and ADG. The relative abundance of *unclassified_Ruminococcaceae* and *Clostridia_UCG-014* was significantly positively correlated with serum GSH-PX content, and negatively correlated with serum MDA content (Figure 6A). The relative abundance of *Butyricicoccus* was significantly negatively correlated with the expression levels of *COX-2, IL-6* and *IL-10* in the jejunum. The relative abundance of *Lactobacillus, Bacteroides, Parabacteroides, unclassified_Ruminococcaceae*, and *Butyricimonas* were positively correlated with the expression levels of *COX-2, mPGES-1, IL-1β* and *IL-6* in the jejunum (Figure 6B).

## DISCUSSION

While the adoption of a high density feeding approach may offer potential benefits in terms of cost reduction and income generation, its implementation can lead to adverse changes in the behavior, physiology, metabolism, and health of broiler chickens, culminating in the manifestation of stress responses (Jones et al*.,* 2005; Najafi et al*.,* 2015; Evans et al*.,* 2023). When the physiological stress response mechanism is activated in broiler chickens under stress, it results in changes in both the immune system and the blood biochemical parameters. This causes the broilers to expend additional energy to overcome the negative effects of stress, which reduces their growth performance (Sugiharto, 2022). The results of this study align with previous research, demonstrating that as the feeding density increased, the body weight of broilers decreased. Additionally, a decrease in the ADFI and ADG of broilers has been noted when the feeding density exceeded 20 broilers/m^2^ (Jobe et al*.,* 2019; Li et al., 2019). During the experimental period, the HD group had a significantly reduced ADFI compared to the ND group from d 0 to 21. Similarly, the HD group had a lower body weight gain compared with the ND group from d 22 to 42. Additionally, the FCR of the HD group was higher than that of the ND group. However, there were no significant differences observed in the ADFI of broilers throughout this growth phase. Based on our findings, it was suggested that HD stress exerts a more significant influence on productivity within the period of 22 to 42 d as opposed to the initial 0 to 21 d time. Therefore, we performed a detailed analysis of 22 to 42 d, and found that HD stress had a significant effect on the production performance of 28 to 42 d of rapid growth period, and in addition, the addition of AEE to the diet significantly alleviated the decline in the performance of HD stressed broilers at this stage. This result supports the hypothesis that the incorporation of AEE into the diet is an efficacious method for mitigating the detrimental consequences of HD stress on overall growth performance.

The increase in feeding density of broiler chickens often results in reduced cleanliness in the rearing environment with a consequent increase in stress (Li et al*.*, 2019; Li et al*.,* 2023). The level of GSH-PX and SOD and their contribution to the T-AOC constitute clear indicators of the status of the antioxidant defense system. The MDA concentration reflects the magnitude of lipid peroxidation taking place within the organism, and serves as an indirect measure of the level of tissue peroxidation-induced damage (Kong et al*.,* 2022; Ina et al*.,* 2023). In this study, HD stress decreased the activity of GSH-PX and SOD, reducing the T-AOC as measured in the serum of broilers, and increasing the MDA content, confirming that high density feeding causes oxidative stress damage in broilers, constituting one of the underlying reasons for the reduced growth performance observed in broilers subjected to HD stress.

The administration of AEE as a dietary supplement, however, resulted in elevated concentrations of GSH-PX and increased the T-AOC in the serum of broilers exposed to HD stress for 28 and 35 d, while concurrently reducing the levels of MDA on d 28, 35, and 42. The study findings indicated that the inclusion of AEE in broiler diets effectively enhanced the antioxidant capacity while concurrently mitigating the oxidative stress induced by HD. The findings presented herein exhibit resemblance to prior research conducted on AEE, as documented in the some studies (Zhang et al*.,* 2021). Thus, the pharmacological efficacy of AEE has been demonstrated in enhancing the antioxidant capacity of the organism and mitigating the detrimental effects of HD stress in broiler chickens.

The preservation of characteristic intestinal morphology and the maintenance of an intact intestinal barrier are of utmost importance for the efficient digestion and absorption of nutrients, as well as the prompt elimination of detrimental bacteria within the intestinal tract (Goo et al*.,* 2019; Gilani et al*.*, 2021). Under normal conditions, longer and broader villi facilitate nutrient absorption in the intestine, while the extent of crypts indirectly indicates the pace of new intestinal cell generation and the rate of metabolism of intestinal substances (Zhang et al*.,* 2020). The evidence obtained from our experiments has confirmed that HD stress markedly compromises the integrity of the intestinal system. In addition, several experiments have provided evidence demonstrating a direct correlation between intestinal integrity and fluctuations in productivity gains and losses (Db et al., 2023). This observation is congruent with the outcomes obtained from productivity analyses, supporting the idea that the productivity of individuals may be seriously compromised as a consequence of intestinal harm caused by HD stress.

The tight junction proteins, claudin, occludin, and ZO-1, are crucial factors in preserving the integrity of the gastrointestinal tract. These proteins play a pivotal role in establishing the physical barrier of the intestinal epithelium and maintaining its selective permeability (Liu et al*.,* 2023a). In addition, a considerable body of research has demonstrated that HD stress has a negative impact on the healthy development of the broilers' intestinal tract and hinders the expression of vital genes, such as *occludin* and *ZO-1*, in the intestinal mucosa (Yu et al*.,* 2021; Varasteh et al*.,* 2015; Zhang et al*.,* 2017). Consistent with these results, our data shows that HD stress seriously damaged villus morphology in the jejunum by decreasing VH and V/C, increasing CD, and decreasing the expression of tight junction genes in the jejunum of broilers, dietary supplementation with AEE resulted in a significant elevation in VH and V/C in broilers, while concurrently reducing the CD of the jejunum. However, AEE supplementation had only a limited influence on the expression of tight-junction genes of the jejunum. Consequently, these outcomes lead us to consider whether the enhancement of intestinal well-being through AEE could be attributed to its anti-inflammatory properties.

Disruption of the intestinal barrier is widely considered to increase permeability, allowing the migration of both beneficial intestinal bacteria and harmful pathogens into the lamina propria. This initiates intestinal inflammation and can give rise to serious complications like systemic infection (Vancamelbeke and Vermeire, 2017; Citi, 2018). Cyclooxygenase enzymes (**COXs**) play a significant role in the arachidonic acid-mediated synthesis of prostaglandins, and elevation in the expression of *COX-2* and increased production of prostaglandin E2 (***PGE2***) have been implicated in the inflammatory cascade. The enzyme, *mPGES-1* regulates the critical terminal rate-limiting step in the synthesis of *PGE2*, thus controlling its biological function. These consecutive occurrences lead to the activation of the pro-inflammatory cytokines, *IL-1β, IL-6,* and *TNF-α*, ultimately resulting in the deterioration of the intestinal epithelial cell barrier and significantly contributing to the pathogenesis of inflammatory bowel disease (Lugo et al*.,* 2007; Sun et al*.,* 2021; Li et al*.,* 2022). Multiple studies have documented that diverse inflammatory cytokines, such as *IL-1β* and *TNF-α*, possess the ability to modify the structural integrity of the intestinal barrier. This disruption has been proposed as a plausible mechanism underlying the emergence of intestinal ailments in broilers subjected to HD stress. (Dai et al*.,* 2022). In this study, the addition of AEE to the feed substantially reduced the expression of *COX-2, mPGES-1, IL-1β*, and *IL-6* within the jejunal tissues of broilers subjected to HD stress. The findings of this study indicate that AEE potentially exerts an anti-inflammatory effect through inhibition of a crucial synthetase involved in the metabolic pathway of arachidonic acid. Some studies have confirmed the ability of anti-inflammatory agents to resist inflammation by inhibiting the arachidonic acid/*COX-2* pathway (Yu et al*.,* 2023). Our data indicate that exposure to HD stress has the potential to activate the arachidonic/*COX-2* acid pathway, thereby inducing the release of inflammatory factors, which subsequently disrupts the intestinal barrier integrity. This disruption exacerbates the oxidative stress response in broilers resulting in a considerable decline in productivity. However, our results clearly show that the incorporation of AEE into the feed can counteract these undesirable consequences by inhibiting *COX-2* activation and lowering the HD stress level.

The determination of the cecal microbiome of chickens and the composition of the intestinal microbiota affords great potential for understanding how it interacts with and influences the host's physiological condition (Wu et al*.,* 2023). The intestinal microbiota governs the immune functionality of the organism while simultaneously upholding the integrity of the intestinal barrier. When the intestinal microbiota are in harmony, they represent a well-balanced ecosystem; any alteration in bacterial levels or composition, regardless of specific abnormalities, culminates in dysregulation of the intestinal ecosystem, triggering inflammation and exerting a deleterious influence on the health and well-being of broiler chickens (Kierończyk et al*.,* 2020; Song et al*.,* 2023; Zhang et al*.,* 2023). Several studies have indicated that HD stress can disturb the equilibrium of the intestinal microecosystem (Sugiharto and Ranjitkar, 2019; Wu et al*.,* 2019, 2023), and we confirmed in this study that exposure to HD stress disrupts the microbial homeostasis in the cecum of broilers, leading to a decrease in α-diversity and modifications in β-diversity. The Chao and Shannon indices of the HD group exhibited statistically significant reductions compared to the ND group, but the addition of AEE to the diet restored the α-diversity in the HD group, suggesting that AEE contributed to the establishment of a more resilient microbial community (Zhang et al*.,* 2018).

The predominant bacterial phyla in the intestinal microbiota of poultry consist of Firmicutes and Bacteroidetes (Wen et al*.,* 2021). In the present investigation, Firmicutes and Bacteroidetes collectively represented over 95% of the total phylum composition. Under HD stress, Firmicutes were increased compared with the ND group, while the numbers of Bacteroidetes in the HD group were lower than in the ND group. Studies have shown that Firmicutes have a negative impact on growth performance and intestinal barrier function, while Bacteroidetes have a positive impact (Dai et al*.*, 2022). At the genus level, there was a significant decrease in the relative abundances of *Bacteroides, Faecalibacterium*, and *Phascolarctobacterium* in the HD group. *Bacteroides* play a pivotal role in carbohydrate fermentation and exert inhibitory effects on pathogen colonization. Insufficient numbers of *Bacteroides* in the gut potentially contributes to the onset of inflammatory bowel disease (Stojanov et al*.,* 2020; Yu et al*.,* 2020), *Faecalibacterium* and *Phascolarctobacterium* are known to synthesize abundant amounts of short-chain fatty acids, particularly butyric acid and propionic acid, which play a crucial role in preserving the integrity of the intestinal barrier and modulating the immune response (Zhou et al*.,* 2023; Jing et al*.,* 2023). The observed outcomes could potentially be attributed to variation in the populations of *Bacteroides, Faecalibacterium*, and *Phascolarctobacterium* among the groups, which can lead to inflammatory bowel disease and a decline in growth performance among broilers subjected to HD stress. However, the incorporation of AEE in the diet of the HD+AEE group led to a substantial rise in the prevalence of *Phascolarctobacterium*. Research has demonstrated that supplementation with AEE can enhance the overall diversity of the intestinal bacterial population (Ma et al*.,* 2018). In an investigation of cecal microorganisms in mice, it was observed that AEE reversed the decline in microbial diversity resulting from hyperlipidemia induced by a high fat diet (**HFD**). Furthermore, AEE elevated the proportion of lactobacilli, suggesting that it mitigated the adverse effects of a HFD on the structure of the intestinal microbial community by enhancing the diversity of intestinal microorganisms, thereby promoting intestinal health (Ma et al*.*, 2018). This study's findings revealed a similarity with their research, indicating that dietary AEE mitigated the negative effects of HD stress through its influence on the composition of the intestinal microbiota. The LEfSe analysis revealed that the HD+AEE group was enriched in *Anaerofustis, Anaerofustaceae, CHKCI002, Actinobacteriota* and *Coriobacteriales*. Numerous studies demonstrated that *Anaerofustis* and *Actinobacteriota* played key roles in immune regulation and maintaining intestinal homeostasis. These bacteria have been unequivocally confirmed as necessary contributors to the maintenance of intestinal health (Goel et al*.*, 2022; Xi et al*.,* 2022).

In order to deepen the understanding of the function of the gut microbiota in growth performance and intestinal inflammation, we measured the serum antioxidant enzyme activity and the level of inflammatory cytokines. We found a significant association between the cecal microbial composition and broiler growth, particularly at the genus level. A strong positive correlation exists between growth performance and the relative abundance of lactobacilli. The presence of *Lactobacillus* in the intestinal tract has been observed to diminish the adhesion of pathogenic bacteria, such as *Escherichia coli* and *Salmonella,* to the intestinal wall. Moreover, it has been found to have a noteworthy impact by enhancing growth performance and preserving intestinal health in poultry (Wang et al*.,* 2017; Jian Wang et al*.*, 2021). The growth performance exhibits a negative correlation with the relative abundance of *Bacteroides* and *Faecalibacterium*, which aligns with previous research (Hu et al*.,* 2020). Our findings clearly indicate that prolonged exposure to HD stress induces major alterations in the gut microbiota of broilers, and supplementing the feed with AEE enhances the composition of the cecal microbiota, preserves the integrity of the intestinal barrier and sustains normal immune responsiveness.

## CONCLUSIONS

The results of our study unequivocally show that HD stress caused by high stocking feeding of broilers can lead to a decline in growth performance and antioxidant capacity. Additionally, it adversely affects intestinal morphology and expression of tight-junction proteins, and triggers intestinal inflammation. Notably, there were significant changes in the microbial populations, marked by a substantial decrease in *Faecalibacterium* and *Phascolarctobacterium*. However, the inclusion of AEE in the diet alleviated these HD stress-related alterations in the intestinal microbiota. This dietary intervention also showed promising effects in enhancing antioxidant capacity and mitigating intestinal inflammation, by reducing the expression of COX-2, improving intestinal integrity and enhancing growth.

## Appendix Supplementary materials

Image, application 1

## Footnotes

Supplementary material associated with this article can be found, in the online version, at doi:10.1016/j.psj.2024.103825.
