# Supplementary material for: Effect of dietary aspirin eugenol ester on the growth performance, antioxidant capacity, intestinal inflammation, and cecal microbiota of broilers under high stocking density
Source: Poult Sci. 2024 May 11;103(7):103825. doi: 10.1016/j.psj.2024.103825 (PMC11131080; doi:10.1016/j.psj.2024.103825)
Supplement: Supplementary file 1 [file mmc1.docx]

**Supplementary Material**

**Table S1.** Effect of AEE on growth performance of HD broilers.

| Items | Group | | | | SEM | P-value |
| --- | --- | --- | --- | --- | --- | --- |
|  | ND | HD | ND+AEE | HD+AEE |  |  |
| 0-21d |  |  |  |  |  |  |
| BW,g | 654.70 | 634.22 | 651.69 | 639.67 | 4.69 | 0.38 |
| ADFI,g | 41.02^ab^ | 39.57^b^ | 41.86^a^ | 40.42^ab^ | 0.35 | 0.02 |
| ADG,g | 31.18 | 30.20 | 31.03 | 30.46 | 0.22 | 0.38 |
| FCR | 1.32 | 1.31 | 1.35 | 1.33 | 0.01 | 0.48 |
| 22-42d |  |  |  |  |  |  |
| BW,g | 2527.83^a^ | 2325.28^c^ | 2531.49^a^ | 2406.08^b^ | 22.07 | ＜0.01 |
| ADFI,g | 153.61 | 150.95 | 152.88 | 150.22 | 0.62 | 0.18 |
| ADG,g | 89.20^a^ | 80.53^c^ | 89.53^a^ | 84.12^b^ | 0.92 | ＜0.01 |
| FCR | 1.72^bc^ | 1.86^a^ | 1.71^c^ | 1.78^b^ | 0.02 | ＜0.01 |

ND, normal stocking density fed basal diet; HD, high stocking density fed basal diet; ND+AEE normal stocking density fed basal diet supplemented with 0.01% AEE; HD+AEE high stocking density group fed basal diet supplemented with 0.01% AEE. BW, body weight; ADG, average daily gain; ADFI, average daily feed intake; FCR, feed conversion ratio (feed: gain, g: g).

^a,b,c^Means within a row with no common superscript differ significantly (n = 12, *P* < 0.05)

**
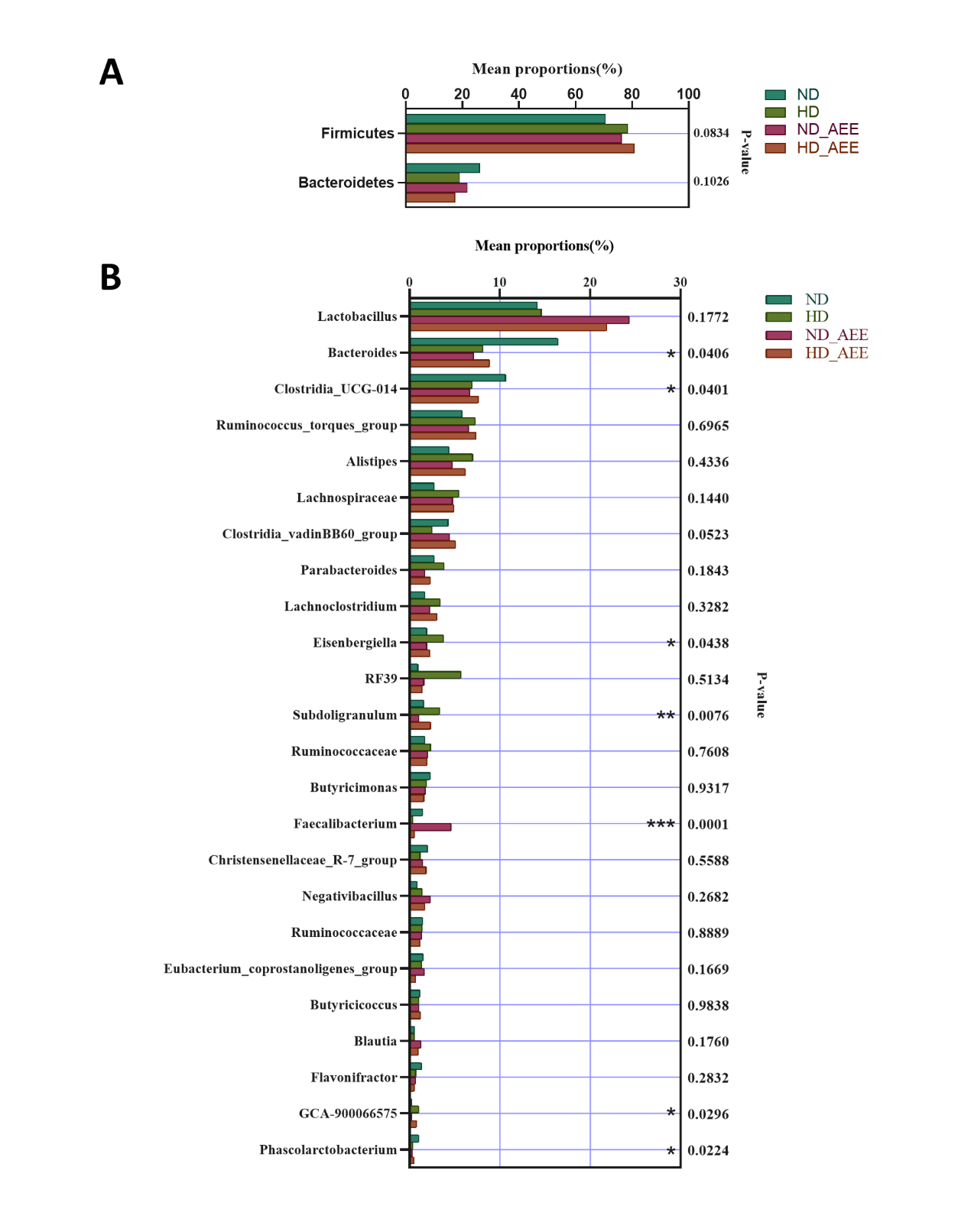
**

**Figure S1. Significant differences in bacteria at phylum and genus level. (A) phylum level.**  (B) genus level. ND normal stocking density fed basal diet; HD high stocking density fed basal diet; ND＋AEE ND, normal stocking density fed basal diet; HD, high stocking density fed basal diet; ND+AEE normal stocking density fed basal diet supplemented with 0.01% AEE; HD+AEE high stocking density group fed basal diet supplemented with 0.01% AEE. “^*^” indicates statistically significant difference (^*^*P* < 0.05, ^**^*P* < 0.01 and ^***^*P* < 0.001)
